# Use of Virtual Reality in Interdisciplinary Multimodal Pain Treatment With Insights From Health Care Professionals and Patients: Action Research Study

**DOI:** 10.2196/47541

**Published:** 2023-11-10

**Authors:** Darcy Ummels, Elise Cnockaert, Inge Timmers, Marlies den Hollander, Rob Smeets

**Affiliations:** 1 Department of Rehabilitation Medicine Care and Public Health Research Institute, Faculty of Health, Medicine & Life Sciences Maastricht University Maastricht Netherlands; 2 Department of Rehabilitation Science Faculty of Medicine and Health Sciences Ghent University Ghent Belgium; 3 Pain in Motion International Research Group Maastricht Netherlands; 4 Department of Medical and Clinical Psychology Tilburg School of Social and Behavioral Sciences Tilburg University Tilburg Netherlands; 5 Adelante Zorggroep Hoensbroek Netherlands; 6 CIR Clinics In Revalidatie Eindhoven Netherlands

**Keywords:** virtual reality, interdisciplinary multimodal pain treatment, chronic pain, pain, rehabilitation, digital health, physiotherapy, occupational therapy, physical therapy

## Abstract

**Background:**

Chronic pain is a widespread global health problem. Interdisciplinary multimodal pain treatment (IMPT) is a treatment option for people with chronic pain. Virtual reality (VR) could be used to broaden IMPT programs. However, despite the advantages of VR, it is rarely used in daily clinical practice.

**Objective:**

This research aimed to explore how, when, and with whom VR can be used meaningfully during IMPT, either as an addition or substitution as a component of IMPT.

**Methods:**

This research used an action research design to help health care professionals and patients learn how, when, and with whom they can use VR. Data were collected through reflection sessions with health care professionals and semistructured interviews with patients in 2 specialized centers that provide IMPT for chronic pain. Two researchers performed direct content analyses.

**Results:**

In total, 4 physiotherapists, 1 occupational therapist, 3 psychologists, and 22 patients participated in this research. Three iteration cycles, including 9 reflection sessions and 8 semistructured interviews, were performed. Both health care professionals and patients considered VR to be useful in therapy as an addition but not a substitution. VR was used as a diagnostic and intervention tool with all patients at the rehabilitation center or home. VR was used to gain insight into patients’ pain beliefs, cognitions, and irrational cognitions about additional damage and physical abilities. Considering VR as an intervention tool, the health care professionals had 3 goals: balancing relaxation and competition, grading activities, and exposure in vivo.

**Conclusions:**

VR could be a valuable addition to IMPT for both patients with chronic pain and health care professionals. More research should be performed to assess the additional effects of VR on patients’ participation in daily life.

## Introduction

Chronic pain is a widespread global health problem. Pain is considered chronic when it persists beyond the expected healing period [1]. At present, 1 in 5 European adults experience chronic pain, of which about a third experience severe pain [2]. Chronic pain can have a significant influence on an individual’s life. It may impair their ability to participate in daily life, interfere with their ability to work, and affect relationships and self-esteem [1]. Research shows chronic pain is one of the most prominent causes of lifetime-experienced disability during daily life, even more so than cardiovascular diseases, diabetes mellitus, or cancer [3]. Consequently, chronic pain has estimated direct and indirect health care costs of €20 million (US $21.24 million) annually in the Netherlands, higher than those of several chronic diseases [4,5].

Interdisciplinary multimodal pain treatment (IMPT) is an option for people experiencing chronic pain. This program does not necessarily aim to reduce pain; instead, it aims to optimize daily life functioning and participation by addressing biomedical, psychological, and social factors contributing to chronic pain and its associated disabilities [6]. The interdisciplinary team responsible for this treatment spans the biopsychosocial spectrum, consisting of a physiatrist, physical therapist, psychologist, and often an occupational therapist. Patients with chronic pain who undergo an IMPT program show considerable improvement in physical and psychological well-being [6]. Nowadays, IMPT primarily involves a combination of physical and psychosocial treatment methods, including emotional awareness and expression therapy, pain neuroscience education, acceptance and commitment therapy (ACT), graded activity, exposure in vivo, and experiential learning through physical training [7]. These treatment modules are provided in gyms or consulting rooms using the physical attributes (eg, balls and dumbbells) and training devices (eg, treadmills) available at the treatment facilities. This limits the performance of specific activities (eg, playing tennis and gardening). Furthermore, some modules, such as exposure in vivo and physical training, require patients to practice at home to establish sufficient training intensity and generalization to daily life. However, the necessary attributes are rarely available in a home environment. Virtual reality (VR) could help to broaden the scope and application of physical and psychosocial treatment modules.

VR allows a user to view and interact with a simulated 3D world. For example, when playing tennis, a user sees a tennis court and a ball coming toward them. To hit the ball, the user has to perform a physical movement. VR could be a meaningful addition to physical treatments by creating situations that are impossible to provide in available training facilities, adding variety, enhancing generalization, and providing insight for both health care professionals and patients into patient behavior (eg, being avoidant). Furthermore, VR often makes physical activity more enjoyable and can be applied in the patient’s home or work environment. Several articles and systematic reviews have been conducted on clinical VR studies, showing that VR reduces experienced acute and chronic pain and kinesiophobia and enhances patient satisfaction and general health status [8-13].

However, despite its advantages, VR is rarely implemented as a component of IMPT. This aligns with the trends of other eHealth apps. Several reports on the use of eHealth methods describe the transition from the pilot phase to implementation, highlighting that upscaling to daily clinical practice remains a bottleneck [14,15]. Barriers to and facilitators in implementing eHealth into daily clinical practice include the complexity of the eHealth tool, health care professionals’ and patients’ digital health literacy, and the perceived benefits [16-19]. Therefore, before implementing a new tool as part of IMPT, exploring when, how, and for whom it can benefit the clinical care process is vital. It is crucial to determine in which phase of the clinical care process a tool can be used (ie, diagnostic, therapeutic, or aftercare); which patients have the physical capacity, emotional well-being, and (digital) literacy skills to use it; and whether VR also can be used in a patient’s home. Therefore, to facilitate optimal implementation, this research aims to explore how, when, and with whom VR can be used meaningfully during IMPT. It uses an action research design to address the following research questions: (1) how do health care professionals and patients use VR as an addition or substitution in IMPT? (2) What are health care professionals’ and patients’ experiences of using VR as an addition or substitution in IMPT?

## Methods

### Overview

**Figure 1:**
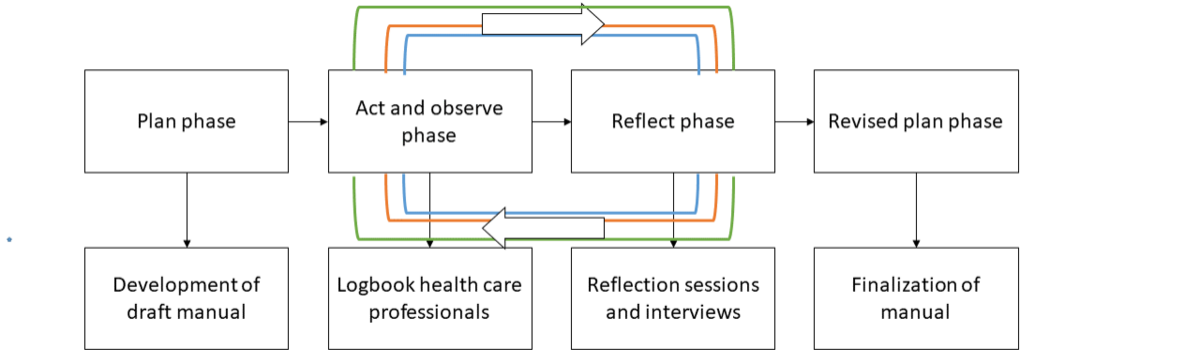
Schematic overview of the design, including data collection. Blue indicates the first round (n=7 health care professionals and n=7 patients), red indicates the second round (n=7 health care professionals and n=6 patients), and green indicates the third round (n=7 health care professionals and n=8 patients).

This research used an action research design. Health care professionals and patients at 2 rehabilitation centers providing IMPT had the opportunity to experience, reflect, and learn how, when, and with whom they could use VR in the program. The action research design consisted of 4 phases: plan phase, act and observe phase, reflect phase, and revised plan phase. Figure 1 presents a schematic overview of this design.

The research team developed a draft manual in the plan phase. The content of the draft manual was based on an earlier developed manual on how to use activity trackers in patients with chronic pain [20]. The content consisted of an introduction to VR, an introduction to the setting and context of the rehabilitation center, the possibilities of VR, and the use of VR in therapy.

In the act and observe phase, health care professionals used VR in daily clinical practice. The act and observe phase lasted for at least 4 to 6 weeks, during which part of the data collection occurred.

The remainder of the data collection was completed during the reflect phase. Health care professionals further developed the draft manual to suit their specific contexts and needs based on their and their patients’ experiences. The act and observe phase and reflect phase were iterative cycles and were planned to be repeated until there was no need for the manual to be further developed. The manual was finalized in the revised plan phase.

### Ethical Considerations

This study was approved by the local ethics board Medical Ethical Committee METC Zuyderland (METCZ20220030).

### Setting and Context

This study was performed in 2 specialized rehabilitation centers that provide IMPT for patients with chronic musculoskeletal pain: the Centre for Integral Rehabilitation (CIR, locations Eindhoven and Velp) and the Adelante Healthcare group (Adelante, location Hoensbroek). Two locations of CIR participated and 1 location of Adelante participated. In both centers, an interdisciplinary team consisting of a physiotherapist or occupational therapist, a psychologist, and a physiatrist provided therapy. Both centers provided pain neuroscience education, ACT, graded activity, exposure in vivo, and experiential learning through physical training [7]. CIR also provided emotional awareness and expression therapy. VR was used in all the above-described therapies.

### VR

**Figure 2:**
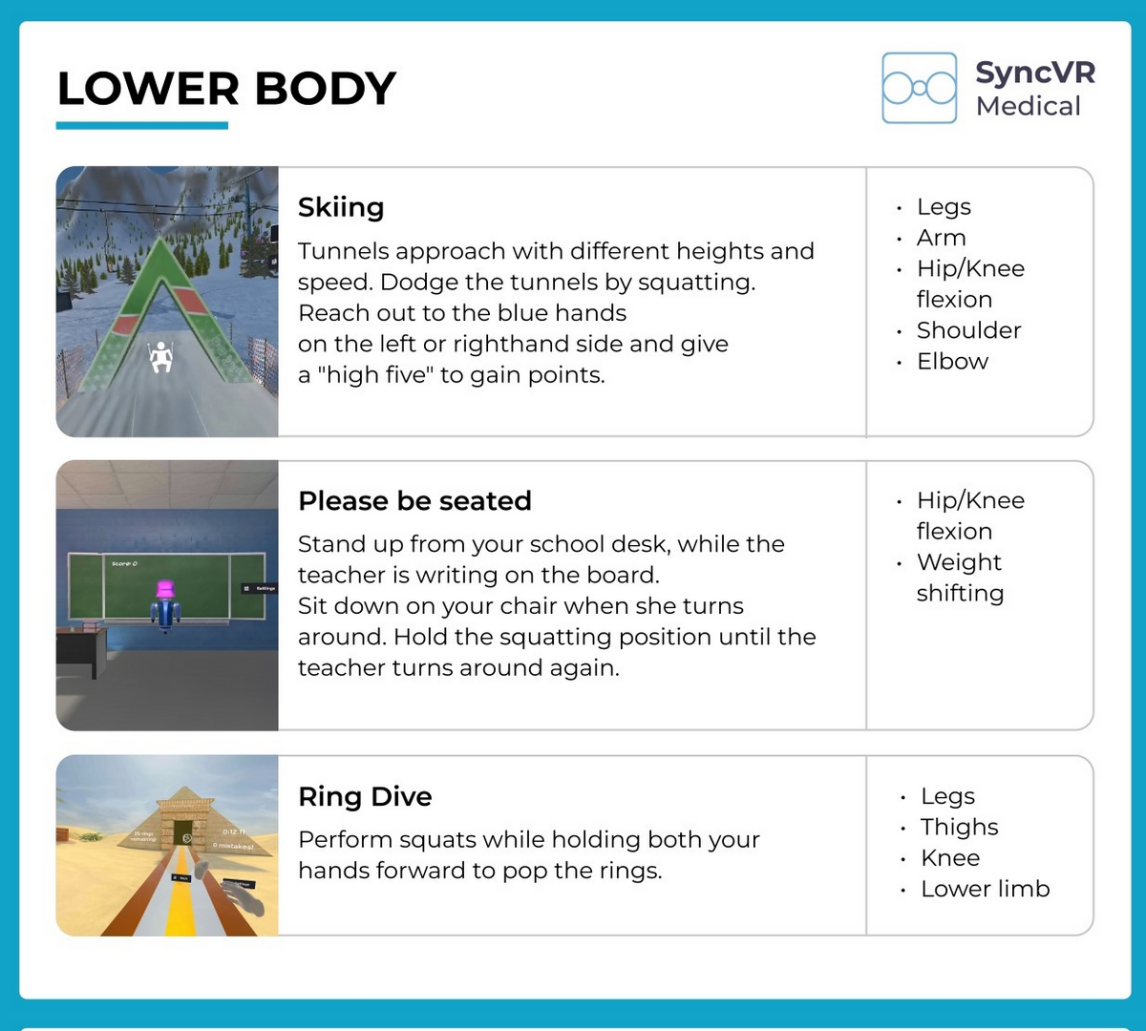
Examples of games for the lower body from SyncVR Fit. Copyright 2022. SyncVR Medical Holding BV [21].

**Figure 3:**
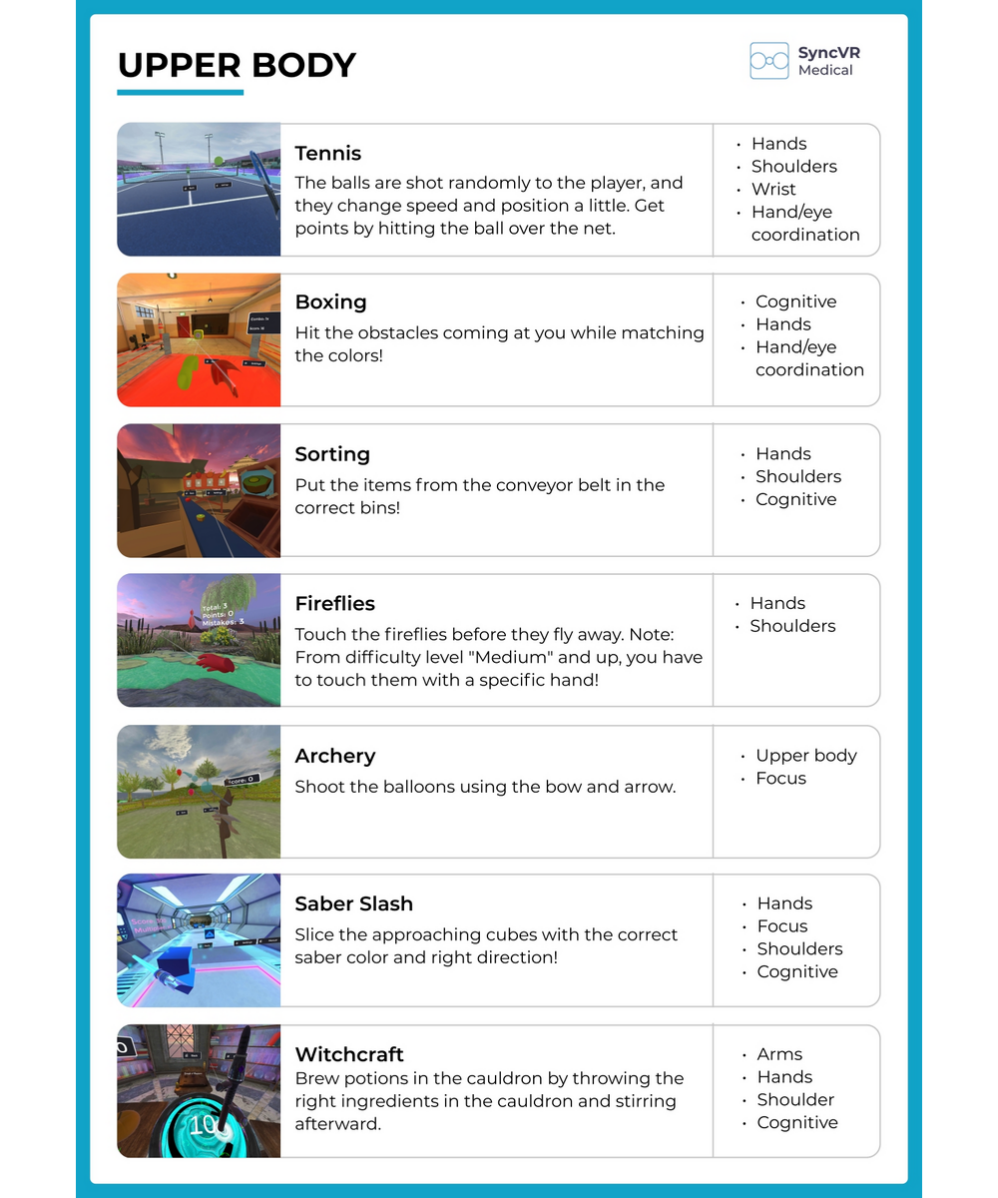
Examples of games for the upper body from SyncVR Fit. Copyright 2022. SyncVR Medical Holding BV [21].

The Oculus Meta Quest 2 VR was used with 2 handheld controllers during this study. The VR software technology SyncVR (Syncv VR Medical [21]) was also used. SyncVR provides various games that can be played with 1 or 2 hands while standing, sitting, or lying down (Figures 2 and 3).

### Participants

Both health care professionals and patients were recruited through the rehabilitation centers. The manager of each rehabilitation center selected the health care professionals using convenience sampling. Health care professionals were included if they were motivated to use VR and could participate for the duration of the study. The health care professionals participated in all phases of the study.

Patients were eligible for inclusion if they received therapy from a participating health care professional and were eligible for blended therapy (ie, use of VR at the rehabilitation center or at home) according to the checklist for blended physiotherapy [22]. The checklist for blended physiotherapy assesses whether patients are eligible for blended therapy. The checklist consists of 8 items such as motivation, safety, equipment, digital skills, health literacy, self-management, time, and financial factors. Patients were selected via convenience sampling. Patients were included if they had a therapy goal that could be (at least partly) achieved using VR. Patients were excluded if they had delirium, dementia, open head or facial wounds, epilepsy, or severe hearing or vision problems. The exclusion criteria were formulated to guarantee the participants’ safety. After being provided with information about the research, patients had the opportunity to consider participation for at least 1 week. Patients participated in 1 cycle of the act and observe and the reflect phases. New patients were included if a new act and observe phase was started. The aim was to include 2 new patients in each act and observe phase for each participating health care professional.

### Data Collection

Data were collected between April and December 2022 using a multimethod approach consisting of reflection sessions with the health care professionals, semistructured focus groups and individual interviews with the patients, and a logbook kept by the health care professionals. Demographic characteristics of the health care professionals and patients were also collected. These included data on the health care professionals’ profession, gender, years of working experience, and years of experience with VR. Data on the patients’ gender, age, diagnosis (categorized according to the International Classification of Diseases-11 [23]), treatment, and weeks of treatment were collected. Furthermore, additional parameters of the participating patients were measured to obtain additional insight into whether specific patient characteristics were important to consider when assessing the feasibility of VR. These included mood (using the Hospital Anxiety and Depression Scale (HADS) [24]), fear of movement (using a shortened version of the Photograph Series of Daily Activities (PHODA) [25]), aerobic capacity (using the 6-minute walk test (6MWT) [26]), reading ability (using the Single Item Literacy Screener (SILS) [27]), and digital skills (using the Quickscan Digital Skills assessment [28]). The HADS subscales range from 0 to 21, with a higher score representing a greater likelihood of anxiety or depression. PHODA ranges from 0 to 100, with a higher score representing a greater likelihood of fearing movement. In the 6MWT, walked distance is calculated as a percentage of the average physical capacity of a population of matched age, gender, and body weight. SILS ranges from 1 to 5, with a higher score representing lower reading abilities. Quickscan ranges from 1 to 3, with a higher score representing better digital skills. These questionnaires and tests were used to assess whether certain patient characteristics are important in determining how, when, and with whom VR can be used during IMPT.

### Reflection Sessions With the Health Care Professionals

After each act and observe phase, a reflection session was held with the participating health care professionals of each center. These were held separately due to practical limitations (ie, different work schedules). These reflection sessions aimed to encourage participants to elaborate on how VR was used and to share experiences of its feasibility and use in daily clinical practice. The research team supported these reflection sessions by leading them and ensuring that the draft manual and clinical reasoning process were discussed. The research team improved the draft manual by adapting it to the health care professionals’ experiences. These group sessions took place either at the rehabilitation center or digitally via Microsoft Teams (Microsoft Corp). They lasted 45 to 60 minutes and were audio-recorded.

### Semistructured Focus Group and Individual Interviews With the Patients

After each act and observe phase, a semistructured or focus group interview was conducted with the participating patients. The interviews were conducted in person at a convenient location for the patients or digitally via Microsoft Teams. The research team led them. The topic list and interview guide were based on a previously developed framework [20]. This framework was initially developed to assess the feasibility of activity trackers but was adapted to the VR context (“VR” replaced the term “activity tracker”; Multimedia Appendix 1). The interviews lasted 30 to 60 minutes and were audio-recorded. The research team used the information gathered during the interviews and focus groups to improve the draft manual.

### Health Care Professionals’ Logbooks

During each act and observe phase, the participating health care professionals had the option to collect their observations in a logbook. Questions in the logbook were “What was the goal of VR treatment with this patient,” “Was this patient eligible for VR treatment,” “What worked well,” and “What am I going to change during the next treatment?”

### Data Analyses

To analyze the data, the research team transcribed verbatim the audio recordings of the reflection sessions with the health care professionals, the semistructured focus group, and the individual interviews with the patients. They used directed content analyses to analyze all the data. NVivo (version 12; QSR International) was used during the analysis process. The researchers used deductive coding based on the applied framework (Multimedia Appendix 1). When a text fragment was relevant but did not match an existing code, an “other” code was created using inductive coding. Two researchers (DU and EC) coded the first transcript and every fifth transcript thereafter. A third researcher (RS) was consulted if the researchers did not reach a consensus. Descriptive statistics of the health care professionals and patients were presented as medians (range). The scores of the measurement tools (HADS, PHODA, 6MWT, SILS, and Quickscan) were calculated and interpreted according to their guidelines.

## Results

### Overview

The research team performed 3 iteration cycles, including 9 reflection sessions and 8 semistructured focus group interviews.

### Health Care Professional and Patient Characteristics

The research included 4 physiotherapists, 1 occupational therapist, and 2 psychologists (Table 1). In addition, 22 patients (Table 2) participated in the study.

**Table 1:** Characteristics of the health care professionals (n=7).

Characteristics		Values
Gender (male), n (%)		3 (43)
Age (years), median (IQR)		34 (31-43)
**Occupation,** **n** **(%)**		
	Physiotherapist	4 (57)
	Occupational therapist	1 (15)
	Psychologist	2 (28)
Working experience (years), median (IQR)		10 (3-22)
Experience with VR^a^, months, median (IQR)		0 (0-0)
Number of patients with whom VR was used, median (IQR)		2 (1-3)

^a^VR: virtual reality.

**Table 2:** Characteristics of the patients (n=22).

Characteristics			Values
Gender (male), n (%)			10.0 (45)
Age (years), median (IQR)			48.0 (32.0-56.0)
**Diagnoses,** **n** **(%)**			
	Primary pain	2 (9)	
	Cancer pain	0 (0)	
	Postsurgical and posttraumatic pain	5 (22)	
	Neuropathic pain	3 (13)	
	Headache and orofacial pain	0 (0)	
	Visceral pain	0 (0)	
	Musculoskeletal pain	11 (48)	
	Other	2 (9)	
Number of weeks in therapy before starting VR^a^ treatment, median (IQR)			6.0 (6.0-10.0)
HADS^b^ anxiety score, median (IQR)			8.8 (5.3-11.8)
HADS depression score, median (IQR)			9.0 (6.3-12.0)
PHODA^c^ score, median (IQR)			26.5 (20.6-42.9)
6MWT^d^ score, median (IQR)			67% (60.0-74.5)
SILS^e^ score, median (IQR)			1 (1.0-1.0)
Quickscan Digital Skills score, median (IQR)			2 (3.0-3.0)

^a^VR: virtual reality.

^b^HADS: Hospital Anxiety and Depression Scale.

^c^PHODA: Photograph Series of Daily Activities.

^d^6MWT: 6-minute walk test.

^e^SILS: Single-Item Literacy Screener.

Of the 22 patients involved in the study, 10 were willing to participate in an interview. The coding framework was based on a previous framework [20] developed to assess the feasibility of activity trackers. The framework was adapted to the VR context by replacing the term “activity tracker” with “VR” (Multimedia Appendix 1). The categories were instructions, characteristics of the VR device, correct functioning, skills and beliefs, goal of the VR device, and use of the VR device. No categories were added based on the inductive analyses. No third researcher was needed during the analyses to resolve any disagreements. The experiences of the health care professional and the patient clearly varied depending on how the VR device was used. For example, a good experience with a device was closely linked to receiving adequate instructions on using it. Due to this strong link between the “use” of VR and the “experience” of VR, these aspects were described together.

### Instruction

During the reflection session, all health care professionals indicated that they started their sessions by explaining to the patient how the VR device worked (ie, using the controllers). However, the health care professionals set up the VR device before the treatment session because they felt that setting it up with the patient was too time-consuming. Health care professionals expressed that they required good insight into how the device worked to guide the patient. Therefore, they had to play the games themselves. Health care professionals expressed that having a guide with screenshots of the games would have been helpful. Throughout the study, the health care professionals felt increasingly confident in providing instructions as they gained more experience with the VR device. The patients generally indicated that the health care professionals’ technical explanations of VR software and devices were sufficient. However, patients who lacked technical skills or had no previous experience with other game consoles felt that the instructions were too limited:

If you are younger, you might have experience with game consoles, but if you are a little bit older, it would be helpful to provide some more technical information.

Patient

It is important to explain VR very well; they handed me the consoles while I was already wearing VR [glasses]. I would prefer a moment to look at the consoles to see what they are and how and which buttons I have to use. When you are already wearing VR [glasses], you can’t see them [consoles] anymore.

Patient

The health care professionals’ explanations of the goal of VR varied. During the first session using VR, the health care professionals did not explain the goal of using VR. They also did not inform the patient whether they could stop the game or choose another one. Health care professionals chose to obtain information about pain beliefs, fear avoidance beliefs, and irrational cognitions to assess the patient’s physical abilities. The health care professionals feared that if they explained the purpose of VR, the patients would become more vigilant and act differently. During the follow-up (intervention) session using VR, the health care professionals did explain the goal because the patients were already aware of VR:

Okay, and why didn’t you share those instructions?

Interviewer

Well, I didn’t want that he felt I had certain expectations since he is really sensitive to that, especially if he doesn’t meet those expectations.

Health care professional

Patients expressed that they did not need explanations of the goal of using VR. They were not bothered by the fact that their health care professionals initially did not explain the goal of using VR. The patients claimed that if they knew the goal, they would not have achieved the results they had:

They [healthcare professionals] instructed me as little as possible about the goal of VR. Since I would likely focus too much on that goal, I think it was very smart of them [healthcare professionals].

Patient

### Characteristics of VR

Both health care professionals and patients claimed that the VR devices were easy to install and use. They liked the games’ interfaces. However, when health care professionals were asked about their wishes, they said they would prefer some games to be more related to the reality of daily life. For example, they could involve putting dishes in (high) cabinets. This could help patients with the transfer from VR to the real world. Furthermore, all games were in the patients’ primary language (Dutch), except 1, which was in English. For some patients, this dissuaded them from wanting to play the English game. “There was one English game, which wasn’t an option for my patient since she couldn’t understand English” [Health care professional].

The health care professionals and patients reported that they felt everybody, apart from those already excluded from the study, could use VR. They also added an additional exclusion criterion, severe balance issues, to protect the safety of patients. The health care professionals stated that the characteristics of participants (which were available through validated questionnaires used to measure anxiety, depression, fear of movement, physical capacity, reading ability, and digital skills) did not influence whether they introduced the VR device. Patients with limited digital skills required more instruction before using the VR device. Nevertheless, these patients then used the device effectively. However, health care professionals considered the patient’s digital skills and reading ability before providing a VR device to use at home.

Health care professionals mentioned that VR would be suitable for other patient populations, such as those with heart disease or spinal cord injuries. Two patients expressed that they lacked sufficient hand strength to push the console buttons. Patients reported that the VR hardware was comfortable to wear. Two patients experienced dizziness or nausea after using the VR device; this was addressed by using VR for a shorter period in the next session:

I experienced that I could use VR for a maximum of 15 minutes before I got nauseous.

Patient

Some patients indicated that they would like more feedback from the VR device. Many would like some encouragement and motivation from the device. Other patients wanted to receive feedback when they were “overactive.” These findings suggest that VR software should be able to adapt its feedback to the goals and beliefs of each patient.

### Correct Functioning

Health care professionals and patients reported almost no technical problems with the VR devices. Any problems that occurred were managed and mitigated.

### Skills and Beliefs

Health care professionals believed they were sufficiently skilled to use VR without support in their daily practice. The patients indicated that after receiving instructions from the health care professionals, they could use the VR device independently. Both health care professionals and patients believed that VR could add value to daily clinical practice:

I notice the enthusiasm of the patients when they use VR; they are so enthusiastic. They really want to talk about their experience.

Health care professional

### Goal of VR

Throughout this study, the health care professionals discovered they could use VR as both a diagnostic and an intervention tool. As a diagnostic tool, health care professionals used VR to gain insight into their patients’ pain beliefs, fear avoidance beliefs, and irrational cognitions about potential additional damage and physical abilities. This was always central to the first session using VR. The health care professionals identified 3 goals for using VR as an intervention tool: exposure, graded activity, and balancing pacing and competition. During the final act and observe phase, patients were allowed to use VR at home as an intervention tool with the same goals described above:

I want them [the patients] to experience movements which they think can’t perform; by working with VR, they experience they perform that movement.

Health care professional

### Use of VR

In both centers, health care professionals and patients indicated that VR could be used only in individual sessions. In the participating rehabilitation centers, therapy trajectories start with group sessions. Therefore, the point when VR is introduced may differ for each patient. However, in this study, all patients started using VR after roughly 6 weeks (of a 10-week therapy trajectory). The health care professionals indicated that VR theoretically could have been used sooner. However, they all felt they needed time to develop relationships with patients, especially those with anxiety or kinesiophobia.

Patients, however, were divided; on the one hand, some of them felt VR could be used. The patients’ opinions were divided. On the one hand, some of them felt that VR could be used sooner. On the other hand, some patients expressed that they needed time to feel comfortable with their health care professional and the process and would not wish to use VR sooner. Both health care professionals and patients indicated that VR did not interfere with their relationship:

I was really focusing on my emotions and could really experience them during VR. If we would have used VR sooner in the trajectory, I think it would have missed its purpose since I didn’t know how and what to feel during that time.

Patient

Generally, therapy sessions lasted between 45 and 60 minutes. VR was used for 15 to 30 minutes during these sessions. Both health care professionals and patients thought this was sufficient. The remaining part of the session was mostly spent discussing the patient’s experience using VR. The discussion primarily focused on the patient’s experience rather than the results of the game. Furthermore, the health care professional and patient discussed the lessons learned from the experience:

We discussed what he [the patient] was thinking, if he crossed boundaries, and if he discovered new things.

Health care professional

Health care professionals found that some patients would not believe they performed certain activities during VR (eg, squatting or raising an arm). Throughout this study, health care professionals came up with a solution to this; they filmed patients using VR and showed them these recordings. This was a valuable feedback tool. When patients used VR at home, health care professionals experienced some difficulties discussing VR sessions. There was no feedback tool, so health care professionals had to lead the conversation solely based on the patients’ experiences.

The health care professionals claimed that observing a patient using a VR device provided helpful information for their diagnostic and therapeutic processes. Furthermore, they expressed that patients were tasked with performing challenging movements by playing a fun game. Patients also indicated that using VR made physical activity fun and distracted them from their pain and disabilities. Furthermore, they were more focused and less distracted by external stimuli (eg, other patients or noise) when using VR. Regular physical exercises are often perceived as boring and mandatory, a problem that VR could partly solve. Both health care professionals and patients perceived VR as an addition to IMPT rather than a substitution for it:

I had to prevent the footballs from going into the goal, and I was thinking about my back, but I didn’t feel my back at all. I was just playing!

Patient

The games and VR are some kind of trigger to move and behave differently than they are used to in real life.

Health care professional

According to patients, using VR at home meant that they could use and practice with VR whenever they wanted. Furthermore, they could share their experiences and goals with their family members:

Surprisingly, they [his children] had the same feeling as I did. They didn’t like to lose a game; for me, this was a reassurance that I am not the only one who feels like this.

Patient

## Discussion

This study aimed to explore how, when, and with whom VR can be used meaningfully during IMPT. The research questions were (1) how do health care professionals and patients use VR as an addition or substitution in IMPT? (2) What are health care professionals’ and patients’ experiences of using VR as an addition or substitution in IMPT?

In this study, health care professionals used VR as a diagnostic and therapeutic tool. As a diagnostic tool, VR provided new information for health care professionals, including insight into patients’ pain beliefs, fear avoidance beliefs, and irrational cognitions about additional damage and physical abilities. When using VR, patients were not aware of the underlying goal of the exercise; therefore, health care professionals felt they could observe more natural behaviors (eg, persistent or avoidant behavior). When considering VR as a therapeutic tool, health care professionals had several goals such as creating a balance between relaxation and competition, graded activity, and exposure in vivo. VR provided the health care professionals more treatment options. Both health care professionals and patients had positive experiences using VR in daily clinical practice. VR provided health care professionals with additional information for their diagnostic and therapeutic processes. Patients expressed that VR made physical activity fun again, distracted them from their pain and disabilities, and helped them focus more on their own experiences.

Several systematic reviews highlight that VR can improve pain intensity, kinesiophobia, mobility, functional capacity, neuropsychological outcomes, quality of life, and physical sensations [11-13,29-31]. Other systematic reviews outline that VR is not effective for people with chronic pain but is effective for those with acute pain [10,30,32]. However, reducing pain is not the primary goal of IMPT. According to Baker et al [29], the most common VR mechanism is distraction and embodiment; distraction is more relevant in treating acute pain and embodiment in treating chronic pain. Patients mentioned both mechanisms and indicated that VR-elicited distraction could be used during ACT treatments and with patients with pain-related fear. However, the immersion and inherent distraction of the VR environment may prevent health care professionals from testing expectancies or the conscious experience of disconfirming the feared consequences of performing a particular activity or movement [33]. The experienced intensity of pain can be influenced by the activity a patient performs. A fun activity (eg, VR) could provide more distraction than a “boring” activity with the same physical intensity. Patients also mentioned that they wanted feedback when they were “overusing.” However, research has shown that people with chronic pain often incorrectly perceive overuse [34].

This study has some limitations. First, there may have been a selection bias for both health care professionals and patients. The participating health care professionals were likely already open and enthusiastic about the use of VR and eHealth. Therefore, the health care professionals may have been open to experimenting with VR and were not afraid to use it. Only 1 health care professional already had experience using VR. Studies show that if health care professionals have previous positive experience with eHealth, they experience more advantages of other eHealth technologies [35,36]. Health care professionals with previous negative experiences may have refused to participate in this study, which could have resulted in a more positively oriented group of health care providers. However, early adaptors can inspire and guide their colleagues when implementing an innovation. The participating health care professionals may represent such early adaptors in daily practice. Second, the same selection bias may have been present in the patients because the health care professionals were free to choose which patients were included in this study. Therefore, health care professionals may have selected patients open to eHealth or VR. The researchers did not assess the criteria the health care professionals used to select their patients. The results show that all patients had sufficient digital skills, although the clinimetric properties of the questionnaire are unknown. Health care professionals’ experiences suggest that other characteristics, such as patients’ anxiety, depression, fear of movement, and physical capacity, did not influence the instruction and use of VR or its feasibility. Third, only 10 of the 22 patients were willing to participate in an interview. Therefore, the whole scope of patients’ experiences may not have been included. Patients’ reasons for not participating in an interview included the required time investment, illness, or failure to attend the interview.

This study is made more robust by its use of iterative cycles. Iterative cycles gave health care professionals time to reflect on their clinical reasoning and use of VR in their daily clinical practice. By experimenting with VR, therapists could learn how, when, and with whom VR could be used. In addition, by discussing their experiences, they could learn from and inspire each other. Furthermore, the patients’ experiences were anonymously shared with the health care professionals. Credibility and transferability were checked to ensure the reliability of this study. Credibility was ensured by using data triangulation (multiple data sources), researcher triangulation, and method triangulation (multiple data collection methods). Transferability was ensured by providing a comprehensive description of the study population and process.

All patients reported that their technical skills were sufficient to use this VR device. Future researchers could conduct a study in which more patients have no or limited digital skills and limited reading ability. However, patients may not need digital skills to use VR during therapy sessions in a rehabilitation center. In this study, health care professionals set up the VR device for their patients. Therefore, the patients only needed to learn how to play the game. If VR is used at home or as a substitution for regular care, patients may need some digital skills to use the VR device by themselves.

This research exemplifies how to use VR in daily clinical practice with patients with chronic pain undergoing an IMPT program. Further research could focus on whether using VR in daily clinical practice positively affects patients’ participation in daily life. Furthermore, future research could focus on the further development of VR devices. A greater variety of games could be developed, especially those involving the lower extremities, activities based on daily life, and relaxation exercises. Furthermore, devices could give the patient and health care professionals direct feedback. By adding these features, VR devices could be used as a substitution for an IMPT program instead of merely an addition.

In conclusion, both health care professionals and patients with chronic pain had positive experiences with VR during IMPT. VR was used as an addition to IMPT for patients with chronic pain and as a diagnostic tool providing insight into pain beliefs, fear-avoidance beliefs, and irrational cognitions about additional damage and physical abilities. As a therapeutic tool, VR was used to create a balance between relaxation and competition, graded activity, and provide exposure in vivo at the rehabilitation center or at home. VR is not yet a substitution for care. Further research should be performed to establish the effects of VR on patients’ participation in daily life and how VR could be used as a substitution for other treatments.

## Appendix

Multimedia Appendix 1 Topic list based on a previously developed framework [20].

## Abbreviations

6MWT: 6-minute walk test
ACT: acceptance and commitment therapy
CIR: Centre for Integral Rehabilitation
IMPT: interdisciplinary multimodal pain treatment
HADS: Hospital Anxiety and Depression Scale
PHODA: Photograph Series of Daily Activities
SILS: Single Item Literacy Screener
VR: virtual reality
