# Supplementary material for: Use of Virtual Reality in Interdisciplinary Multimodal Pain Treatment With Insights From Health Care Professionals and Patients: Action Research Study
Source: JMIR Rehabil Assist Technol. 2023 Nov 10;10:e47541. doi: 10.2196/47541 (PMC10674140; doi:10.2196/47541)
Supplement: Multimedia Appendix 1 [file rehab_v10i1e47541_app1.docx]

**Appendix 1.**

The topic list and interview guide for the interview were based on a previously developed framework [1]. This framework was originally developed to assess the feasibility of activity trackers but was adapted to Virtual Reality (VR) (the term activity tracker was replaced by VR).

| **Category** | **Subcategory** |
| --- | --- |
| Purchase | Costs of the VR |
|  | Costs of a subscription |
|  | Compensation of healthcare insurance |
|  | Possession of a smartphone |
|  | Possession of a computer |
|  | Available and clear information about the feasibility of the VR |
| Instruction | Required instruction from healthcare professional |
|  | Support |
|  | Required technical skills |
| Characteristics of the VR | Installing and receiving data from the VR |
|  | Measured variables by the VR |
|  | Interface |
|  | Accessibility |
|  | Wearing comfort |
|  | Setting goals |
|  | Complexity |
|  | Feedback |
|  | Robustness |
| Correct functioning | Validity |
|  | Reliability |
|  | Technical problems |
| Skills and beliefs | Beliefs of healthcare professional |
|  | Beliefs of patient |
|  | Skills of therapist |
|  | Skills of patient |
| Sharing data and privacy | Interoperational |
|  | Possibility to share data |
|  | Safely sharing data |
|  | Warrant of privacy |
|  | Insight into data by healthcare professional |
|  | Authorization, authentication, license |
| Goal of the VR | Diagnosis |
|  | Assessment |
|  | Monitor |
|  | Intervention |
| Use of the VR | Implementation in therapy |
|  | Implementation in clinical reasoning |
|  | Interface |
|  | Compliance by healthcare professional and patient |
|  | Setting goals |
|  | Choice of VR |
|  | Discussing data |
|  | Data interpretation |
|  | Feedback technical problems by patients |
|  | Healthcare professional and patient relation from perspective of the healthcare professional |
|  | Healthcare professional and patient relation from perspective of the healthcare patient |
|  | Added value of the VR |
|  | Faith in measurements and measurements procedures |
|  | Length of use |

*Abbreviation: VR; Virtual Reality

1. Ummels D, Beekman E, Braun SM, Beurskens AJ. Using an Activity Tracker in Healthcare: Experiences of Healthcare Professionals and Patients. Int J Environ Res Public Health. 2021;18(10).
